# Polarization conversion in plasmonic nanoantennas for metasurfaces using structural asymmetry and mode hybridization

**DOI:** 10.1038/srep40906

**Published:** 2017-01-19

**Authors:** Peter R. Wiecha, Leo-Jay Black, Yudong Wang, Vincent Paillard, Christian Girard, Otto L. Muskens, Arnaud Arbouet

**Affiliations:** 1 CEMES, CNRS and Université de Toulouse, 29, rue Jeanne Marvig 31055 Toulouse Cedex 4, France; 2 Physics & Astronomy, Faculty of Physical Sciences and Engineering, University of Southampton, Highfield, Southampton SO17 1BJ, United Kingdom

## Abstract

Polarization control using single plasmonic nanoantennas is of interest for subwavelength optical components in nano-optical circuits and metasurfaces. Here, we investigate the role of two mechanisms for polarization conversion by plasmonic antennas: Structural asymmetry and plasmon hybridization through strong coupling. As a model system we investigate L-shaped antennas consisting of two orthogonal nanorods which lengths and coupling strength can be independently controlled. An analytical model based on field susceptibilities is developed to extract key parameters and to address the influence of antenna morphology and excitation wavelength on polarization conversion efficiency and scattering intensities. Optical spectroscopy experiments performed on individual antennas, further supported by electrodynamical simulations based on the Green Dyadic Method, confirm the trends extracted from the analytical model. Mode hybridization and structural asymmetry allow address-ing different input polarizations and wavelengths, providing additional degrees of freedom for agile polarization conversion in nanophotonic devices.

The use of small antenna elements in controlling electromagnetic waves has taken flight with many applications in nanophotonics such as surface enhanced spectroscopy, control of single-molecule emission, enhanced nonlinear optics and optical trapping. Next to single-antennas, their arrangement in metasurfaces has led to many new applications in flat optics, holograms, and nanoscale structural coloring. These devices rely on plasmonic and dielectric nanophotonic elements capable of precisely tailoring the near-field and far-field response, including polarization, magnetic resonances, and chirality. As one of the most elementary examples of nanoantennas, dimers consisting of two closely coupled nanoparticles are of interest to investigate fundamental principles underlying the response of more general composite nanostructures^1^,^2^,^3^. Apart from metallic nanostructures, recently also non-plasmonic dielectric particles turned out to offer an interesting alternative platform with remarkably low losses^4^,^5^. Due to their stronger polarizability, plasmonic particles are nevertheless the first choice as it comes to polarization conversion applications. Recently, dimer nano-antennas in an orthogonal configuration have been investigated in the context of polarization conversion applications^6^,^7^,^8^, energy transfer^9^, metasurfaces^10^,^11^,^12^, and nonlinear light emission^13^. Contrary to linear dimers, L-shaped nanostructures support two bright plasmon modes which are orthogonally polarized when the two antenna arms are of equal lengths. The possibility of exciting a coherent superposition of these orthogonally polarized eigenmodes allows to control the scattering from these antennas. For instance, the polarization of the light scattered by L-shaped antennas consisting of two nanorods of equal length can be adjusted via a proper choice of the antenna morphology or excitation wavelength. In particular, optimal polarization conversion can be obtained in the spectral range between the antenna eigenmodes when the latter are excited with the appropriate phase and amplitude^6^. Systematic experiments have further shown that this process only depends upon the spectral splitting between the antenna eigenmodes in good agreement with a two-oscillator model. In the case of capacitively coupled dimer antennas, the coupling between the antenna arms and therefore the spectral splitting between its eigenmodes can be tuned through the gap width^3^. Next to disconnected dimer antennas, connected V-shaped and L-shaped antennas have also been investigated in several experimental and theoretical works^10^,^14^,^15^,^16^,^17^,^18^,^19^,^20^.

In this work, we investigate polarization conversion (PC) by orthogonal dimer nano-antennas consisting of two nanorods of unequal length. We address the influence of asymmetry, gap spacing and excitation wavelength on polarization conversion. In the first section, we describe PC in L-shaped dimer antennas with an analytical model based on field susceptibilities. In the second section, the linear optical spectra and polarization conversion properties are investigated using single particle optical spectroscopy and the experimental results are compared to extensive numerical modeling using the Green Dyadic Method.

## Polarization conversion in L-shaped antennas: an analytical model

**Figure 1:**
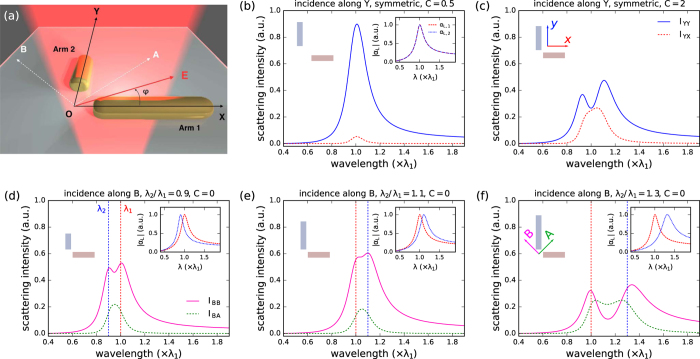
(**a**) Schematic representation of orthogonal antennas consisting of two arms of respective length *L*_1_ and *L*_2_ at (d, 0, 0) and (0, d, 0). Each arm is described by a polarizability tensor *α*_1_(*ω*). *φ* is the angle between the polarization of the incident wave and the (OX) axis. (**b**) Intensity scattered with polarization along (OX) (red dashed line) or (OY) (blue solid line) by an orthogonal symmetrical antenna illuminated under Y-polarization, characterized by a resonance wavelength of both arms *λ*_*sp*,1_ = *λ*_*sp*,2_ = 1000 nm and a coupling constant *C* = 0.5. (**c**) Same for a coupling constant *C* = 2. (**d–f**) Intensity scattered along A (green dashed line) and B (pink solid line) for asymmetric antennas illuminated under B-polarization. Resonance wavelengths are *λ*_*sp*,1_ = 1000 nm and *λ*_*sp*,2_ = 900 nm (**d**), 1100 nm (**e)** and 1300 nm (**f**) with no coupling (*C* = 0).

The optical response of orthogonal nano-antennas is calculated using an analytical approach following earlier studies on coupled plasmonic antennas^21^,^22^,^23^,^24^,^25^,^26^. Figure 1a shows the investigated nanostructure which consists of two identical gold nano-antennas orthogonal to each other, one aligned along (OX) and the other along (OY). We assume that the particles are much smaller than the optical wavelength and use the quasi-static approximation throughout this section. In short, the two antennas are described as Lorentzian oscillators which are coupled via near-field dipole-dipole coupling. By inverting the coupling matrix a solution is obtained for the collective modes of the coupled system.

An optical excitation of the nano-antennas will polarize each antenna arm. In the quasi-static approximation, this induced polarization can be described by a single dipole moment. For instance, for particle (1), we have:


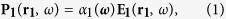


where **P**_**1**_(**r**_**1**_, *ω*) is the dipole induced in particle (1) and **E**_**1**_(**r**_**1**_, *ω*) is the total electric field in the same particle. *α*_1_(*ω*) is the polarizability tensor of particle (1). The latter can be obtained from a numerical fit of the experimental spectra of individual nanoparticles (or antennas with large gaps) or from analytical formulas in simple cases such as ellipsoids or spheres. In the following, we assume that each component of the polarizability tensor has a lorentzian profile and that the width of all optical resonances has the same value *γ*. It is well-known that elongated plasmonic particles support two plasmon resonances called longitudinal and transverse which are selectively excited for an incident optical excitation polarized, respectively, along their long and short axis. In the following, we however do not take into account the transverse plasmon resonance as we investigate polarization conversion in a different spectral range. We further use polarizabilities normalized at the resonance frequency, hence with a constant amplitude |*α*_*L*,*i*_(*ω*_*sp*,*i*_)| = 1, independent of the arm length. The resonance frequencies of an antenna arm being denoted *ω*_*sp*,*i*_, we have for instance for particle (1):


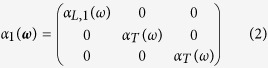


with


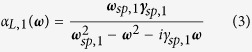


and


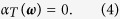


A similar equation holds for particle (2), where the longitudinal polarizability *α*_*L*,2_ is aligned along (OY).

In the framework of the field susceptibility formalism, the self-consistent electric field in particles (1) and (2) is connected to the incident E-field **E**_**0**_(**r**, **ω**) by the following equations:


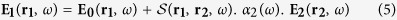



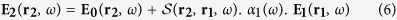


In these equations, 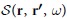 is the Green dyadic function which relates the electric field **E**(**r**, *ω*) induced at ***r*** by a dipole **p**(**r**′, *ω*) located at **r′** by the following equation:


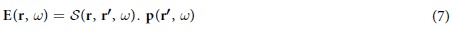


The separation between two nanoantennas being much smaller than the optical wavelength, we only take into account the near-field contribution to the Green Dyadic tensor:


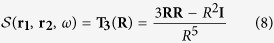


in which **R** = **r**_**1**_−**r**_**2**_, *R* = |**R**| and


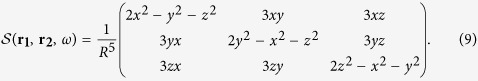


Particles (1) and (2) being respectively located at (*d*, 0, 0) and (0, *d*, 0), **R** = (*d*, −*d*, 0) and the propagator can be written:


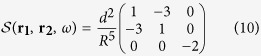


We define *K* = *d*^2^/*R*^5^ = 1/(4(2)^1/2^*d*^3^) as well as a coupling constant normalized to the polarizability at resonance *C* = *K*|*α*_*L*,1_(*ω*_*sp*,1_)|, the latter being dimensionless in CGS units. The total induced dipole can be deduced from the total electric field on each antenna arm:


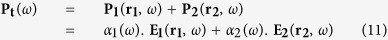


The self-consistent electric fields **E**_**i**_(**r**_**i**_, *ω*) are obtained using a matrix formalism as presented in the Methods section. In the (XY) plane, the components of the total induced dipole parallel (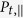) and perpendicular (*P*_*t*,⊥_) to the polarization of the incident wave are:


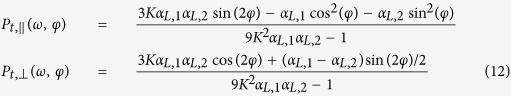


where for clarity the dependence on *ω* was omitted in the expression of the polarizabilities *α*_*L*,1_ and *α*_*L*,2_ and *φ* is the angle between the polarization of the incident electric field and the (OX) axis (See Fig. 1a). The intensity scattered with either polarization is proportional to the square of the modulus of the corresponding total dipole component. The polarization conversion efficiency 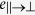 can then be calculated as the ratio of the intensities of the *P*_*t*,⊥_ and 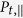 components of **P**_**t**_(*ω*):


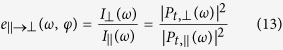


### Case of orthogonal antennas with identical arm lengths

Polarization conversion in symmetric L-shape antennas has been discussed into detail in *Black et al*.^6^ both in the capacitive and conductive coupling regimes. Their optical response is characterized by two orthogonally polarized, bright plasmon resonances. The high energy mode, called *antibonding* (noted *A*), is polarized at 45° whereas the low-energy *bonding* mode (noted *B*) is polarized at 135° with respect to the antenna axes. Light incident along these principal axes does not undergo polarization conversion. Excitation along either the horizontal (OX) or vertical (OY) axes sets up a superposition of the principal A and B modes, resulting in polarization conversion in a region of the spectrum in-between the two modes where these have an unequal phase. In the two-oscillator model presented in ref. 6, an ad-hoc oscillator was associated to each eigenmode of the *coupled* system. On the contrary, in the analytical model presented here, the eigenfrequencies are not an input but an output of the model. The analytical model thus accounts *both* for the hybridization into antibonding and bonding modes and for the polarization conversion in symmetric L-shaped antennas in a self-consistent manner.

For an optical excitation polarized along (OX), the components of the total dipole moment induced are given by


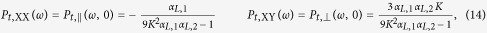


while an excitation along (OY) gives


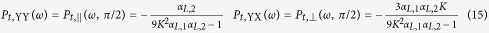


The polarization conversion efficiencies in both cases follow as:


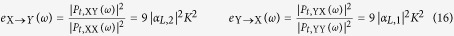


For uncoupled antennas, *K* = 0 and the total dipole on the nanostructure has no component along the direction orthogonal to the incident polarization and therefore no polarization conversion is possible. As expected for a near-field effect, the polarization conversion efficiency is proportional to *K*^2^ ∝ 1/*d*^6^. For weak couplings, only a very small spectral splitting and a weak polarization conversion are observed (Fig. 1b). For stronger couplings, two resonances appear in |*P*_*t*,YY_(*ω*)|^2^ (Fig. 1c), where the resonance at high energy is the A mode whereas the low energy-mode is the B mode. The intensity |*P*_*t*,YX_(*ω*)|^2^ scattered with a polarization perpendicular to the optical excitation, reaches a maximum intensity between the two eigenmodes. In this spectral range, the oscillation of the two eigenmodes is dephased: an incident wave polarized along Y induces two dipole moments with opposite phases at 45° and 135° and the resulting interference is polarized along X. Whereas the dephasing between the eigenmodes becomes closer to *π* when their spectral splitting increases, the intensity scattered along (OX) in this case is expected to remain very small as it is not possible to excite effectively both resonances. The results of the hybridization model are in excellent agreement with the full electrodynamical simulations presented below and with the experimental results given in ref. 6.

### Case of orthogonal antennas with different arm lengths

The two-oscillator model predicts a polarization conversion based on the phase difference of two resonances at different wavelengths. Apart from hybridization through strong coupling, a similar wavelength splitting can be obtained by introducing a structural asymmetry in the antenna by changing the length of one of the nanorods in the dimer.

We now apply the analytical model to the general case of orthogonal antennas with arms of different lengths. We consider that one arm has a fixed resonance wavelength *λ*_*sp*,1_ = 1000 nm while the second arm has a resonance at a variable *λ*_*sp*,2_. In the coupled dipole model we define the asymmetry ratio by these wavelengths, namely *λ*_*sp*,2_/*λ*_*sp*,1_. In order to excite the longitudinal modes of both arms simultaneously, an excitation along 45° or 135° seems adequate in the asymmetric case. Similar to conversion from (OY) to (OX), we can calculate the efficiency for conversion from an incident polarization along the directions of the hybridized antibonding (A: 45°) and bonding (B: 135°) modes of a symmetric antenna (see Fig. 1a). For A-polarized incidence this yields


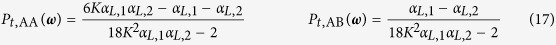


and for B-polarized incidence we get


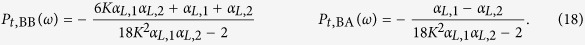


From this we obtain the following A → B and B → A conversion efficiencies:


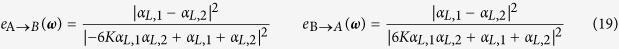


Figure 1(d–f) shows spectra for uncoupled asymmetric dimers with increasing wavelength splittings of comparable strength like in the coupled symmetric case in Fig. 1c. The antennas are driven along 135° (pink, “B”), hence the polarization conversion is along 45° (green, “A”). As expected from the two-oscillator model, PC occurs in the spectral range between the two resonances, similarly to the symmetric antennas. In the following we analyze this case into more detail, and we will be showing that the interplay between asymmetry and coupling always results in a reduced polarization conversion efficiency, if driving the coupled antennas with a polarization along one of the antenna arms.

**Figure 2:**
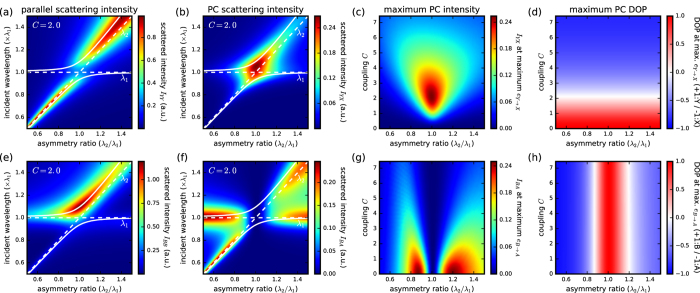
(**a,b**) Spectral intensity maps vs asymmetry ratio *λ*_2_/*λ*_1_, for fixed *λ*_1_ = 1000 nm, for incident polarization along OX and scattered polarization along OX (**a**) and OY (**b**). (**c,d**) Parametric plots showing maximum scattering intensity(**c**) and degree of polarization (**d**) at the wavelength of maximum polarization conversion against asymmetry and coupling strength. (**e,f**) Same as (**a,b**) but for incident light polarization along B and scattered polarizations along B (**e**) and A (**f**). (**g,h**) Same as (**c,d**) for polarization conversion from B to A states.

In Figure 2, we compute the intensity scattered with polarization either along either (OY) (a) *Y (I*_YY_ = |*P*_*t*,YY_(*ω*)|^2^, Fig. 2a) or (OX) (b) *X (I*_YX_ = |*P*_*t*,YX_(*ω*)|^2^, Fig. 2b) as a function of the asymmetry ratio *λ*_*sp*,2_/*λ*_*sp*,1_. Figure 2(a) shows that *I*_YY_ mainly follows the resonance of the vertical arm for large asymmetry, while near *λ*_*sp*,2_/*λ*_*sp*,1_ = 1 it shows a splitting corresponding to the hybridization with the horizontal nanorod. The eigenfrequencies of the coupled system display an anti-crossing behaviour, typical of a system of two coupled oscillators. The intensity scattered along (OX), shown in Fig. 2(b), increases when the two resonances overlap and decreases drastically as their spectral overlap is reduced.

From the spectral dependence of (b), the maximum PC intensity *I*_YX_ can be extracted. Figure 2(c) shows the intensity *I*_YX_ at the wavelength of maximum *e*_*Y*→*X*_, scattered with polarization along (OX) as a function of the asymmetry ratio *λ*_*sp*,2_/*λ*_*sp*,1_ for different values of the coupling *C*. This figure confirms that for symmetric antennas (*λ*_*sp*,2_/*λ*_*sp*,1_ = 1), maximum polarization conversion is obtained for intermediate coupling strength (*C* ≈ 2) where the spectral splitting amounts to the resonance linewidth^6^. When moving away from the symmetric condition, the PC intensity drops rapidly.

Let us now analyze the case of excitation polarized along the direction of the bonding mode of symmetric antennas (135°). Figure 2(e) shows the polarization conserving component *P*_*t*,BB_, which yields the strongest scattering (*I*_BB_) for a resonant excitation of the bonding mode for asymmetry ratios around 1. Away from the symmetric condition, the intensity contribution of the individual nanorod resonances becomes visible as the effect of hybridization is reduced when the modes do not spectrally overlap each other. The intensity scattered with orthogonal polarization *I*_BA_ = |*P*_*t*,BA_|^2^, shown in Fig. 2(f), reaches its highest values away from the symmetric condition and if the system is excited close to one of the resonance wavelengths *λ*_*sp*,1_ and *λ*_*sp*,2_. The parametric map (Fig. 2(g)) shows a significant B → A polarization conversion away from the symmetric configuration and for intermediate asymmetry ratios around 0.85 and 1.2. As discussed above, the B → A configuration does not allow any polarization conversion in the case of symmetric antennas as it corresponds to the excitation of a pure eigenmode.

At the highest polarization conversion intensity however, for both BA as well as YX conversion, the degree of polarization (DOP), defined as


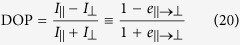


is close to zero, as shown in Fig. 2(d,h). This means that concurrently to the polarization converted scattering *I*_⊥_ a large amount of scattering 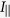 is generated with polarization parallel to the optical excitation. This is indicative of the fact that in all cases, maximum PC intensity requires efficient in- and out-coupling of the optical excitation into both resonances under simultaneous satisfaction of the proper phase relation^6^. For larger splittings, the DOP further increases, however the coupling efficiency to the modes is reduced as the mode splitting increases beyond the resonance linewidth. Both in the symmetric as well as asymmetric case we observe beyond this optimum that a further increase in PC efficiency comes with a decrease of the cross-polarized scattering intensity.

### Transition between hybridization-mediated and asymmetry-mediated polarization conversion

**Figure 3:**
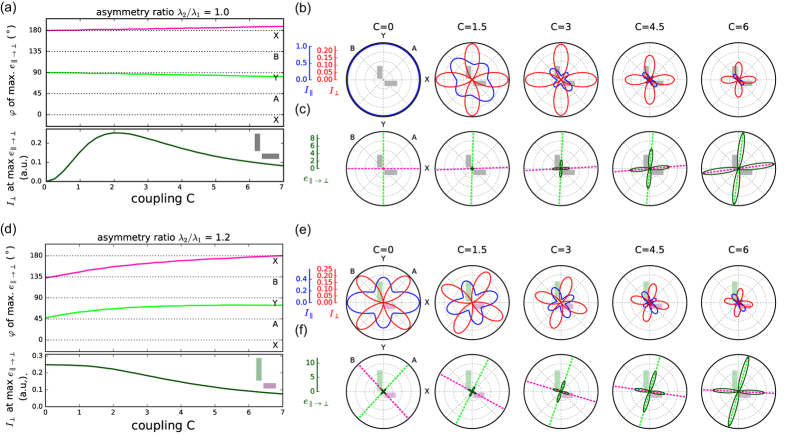
(**a**) Angles of incidence (green, magenta) at which strongest polarization conversion (PC) efficiency 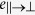 occurs for the symmetric antenna, and corresponding PC scattering intensity *I*_⊥_ (black curve in lower panel) as a function of the coupling strength at the wavelength of maximum PC efficiency. (**b**) Scattering intensity parallel (blue) and perpendicular (red) to the incident polarization at wavelength of maximum 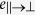. (**c**) Polarization conversion efficiency 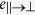 for selected coupling coefficients, angles of maximum PC corresponding to (**a**) are indicated by dashed lines. (**d–f**) Same as (**a–c**) for asymmetric dimer with asymmetry ratio *λ*_2_/*λ*_1_ = 1.2.

The results from Fig. 2 show the general trend that strongly coupled symmetric antennas allow polarization conversion in the XY basis whereas asymmetric antennas without coupling are best suited for PC in the AB directions. However, the parameter maps of Fig. 2 are computed in fixed polarization conditions. These do not take into account the fact that the basis of eigenmodes tilts when combining asymmetry and coupling. To explore this effect further, we investigate the intermediate regime and the transition between the two limiting cases by solving the principal axes in presence of both, asymmetry and coupling. In Fig. 3, we identify for different geometries the polarization angles *φ*_*PC*_ yielding the largest PC efficiency 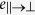 at the wavelength of maximum PC. Figure 3(a) shows the angles of maximum 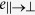 obtained for the symmetric antenna *λ*_2_/*λ*_1_ = 1.0. Corresponding polar plots at five selected values of the coupling strength *C*, shown in Fig. 3(b,c), give the parallel and PC intensities 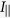 and *I*_⊥_ (b) and the PC efficiency (c).

In the symmetric case of Fig. 3(b), scattering with conserved polarization 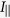 has maxima at 45° and 135° which correspond to the directions of the pure eigenmodes A and B. Conversely, the cross-polarized scattering *I*_⊥_ is maximum for X and Y incident polarizations. The maxima in PC efficiency, shown in Fig. 3a,c, are aligned with the X and Y axis for weak coupling. Due to the highly asymmetric intensities for bonding and antibonding excitation at increasing coupling strength, the angles of maximum PC efficiency 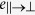 in Fig. 3(d) become slightly offset with respect to the X/Y directions for large couplings, but always remain close to 0° and 90°.

The case of structural asymmetry is illustrated in Fig. 3(d–f) for an antenna with resonance wavelength ratio *λ*_2_/*λ*_1_ = 1.2. In absence of coupling, the pure eigenmodes of this system are now along the X/Y directions. The maximum intensity of polarization conversion is obtained without any coupling for incident polarizations close to the A/B directions as can be seen in Fig. 3(d). Increasing the coupling between the antennas introduces mode hybridization, which gradually tilts the eigenmode angles toward the A/B directions for strongly coupled antennas. As a consequence, the directions of maximum PC efficiency are gradually tilted towards the X/Y basis. Both the maximum cross-polarized intensity *I*_⊥_ in Fig. 3(e) and the PC efficiency 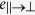 in Fig. 3(f) follow this trend. We note that, whereas the cross-polarized intensity patterns have a perfect four-fold symmetry, the two angles of maximum PC efficiency are generally skewed and not perpendicular to each other due to the asymmetry in the eigenmode intensities contributing to 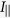.

Note that the PC intensities in Fig. 3(a) and (d) are of similar magnitude, with a slightly larger value in the symmetric case. Thus, it appears that the combination of asymmetry and coupling does not increase the PC efficiency beyond the case of strong coupling in symmetric dimers. This conclusion supports the trends in the parameter maps of Fig. 2(c,g) which were taken at fixed polarizations. In the following, we perform optical spectroscopy experiments on individual antennas and compare our measurements with the results of the analytical model as well as with electro-dynamical simulations.

## Single particle optical spectroscopy experiments

**Figure 4:**
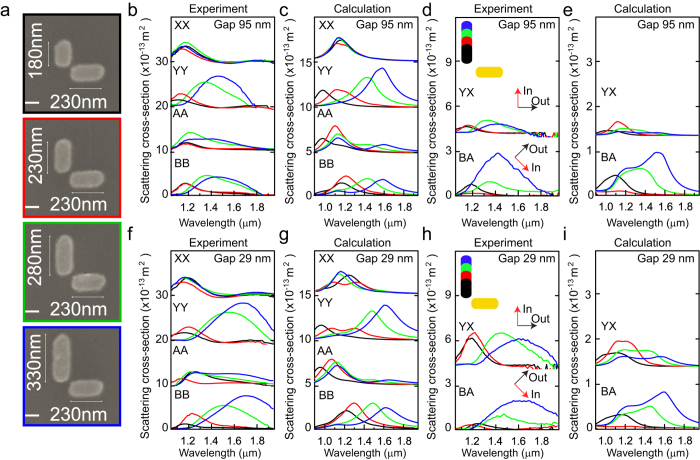
(**a**) SEM images of investigated asymmetric antennas with *L*_1_ = 230 nm and *L*_2_ varying from 180 nm to 330 nm. Experimental (**b,d,f,h**) and simulated (**c,e,g,i**) spectra are shown for antennas with gaps g = 95 nm (**b–e**) and g = 29 nm (**f–i**). (**b,c,f,g**) scattering without polarization conversion for X, Y, A and B incident polarization. (**d,e,h,i**) *σ*_YX_ and *σ*_BA_ polarization conversion spectra respectively from Y → X and B → A.

Orthogonal gold dimer antennas of 30 nm thickness were fabricated using electron beam lithography following methods described in ref. 6. The antennas consist of two perpendicular metallic rods of lengths *L*_1_ and *L*_2_ separated by a gap of width *g*. Figure 4a shows Scanning Electron Microscopy (SEM) images of selected antennas, corresponding to the case where the horizontal nanorod length was kept fixed at *L*_1_ = 230 nm, while the vertical nanorod length *L*_2_ was varied to 180 nm, 230 nm, 280 nm, and 330 nm. All nanorods have a fixed width of 120 nm.

Quantitative measurements of the scattering cross-sections for different polarizations were obtained using Spatial Modulation Spectroscopy^6^. To investigate the effect of different incident polarizations, intensities in the parallel and perpendicular scattering polarizations were determined in both the (X, Y) and (A, B) basis. Figure 4b shows experimental cross sections for orthogonal antennas with a gap of 95 nm, while results for antennas with a gap of 29 nm are shown in Fig. 4f. The antenna size parameters correspond to those of Fig. 4a, as labelled by the colored frames. The parallel polarized scattering cross sections are labelled as *σ*_XX_, *σ*_YY_, *σ*_AA_, and *σ*_BB_, where the first subscript represents the incident polarization and the second the detection polarization.

To interpret these results and go beyond the analytical model, we perform numerical simulations using the Green Dyadic Method (GDM)^27^,^28^ for which the dielectric response of gold was taken from Johnson and Christy^29^. The GDM simulation technique relies on a volume discretization of the nanostructure into a collection of polarizable entities placed on a cubic lattice. The method allows the computation of a generalized field propagator to describe the near-field and far-field optical response of nanostructures of arbitrary shapes placed in complex environments^30^,^31^,^32^. The knowledge of this generalized propagator allows computing the electric field and polarization induced inside the nanostructure by any type of illumination^27^,^28^. The energy radiated in the far-field inside the solid angle defined by the collecting optics is then computed from the polarization distribution created inside the nano-antennas. The contribution from the substrate, which was not taken into account in the analytical model, is fully taken into account by adding to the field-susceptibility of vacuum an additional term accounting for the contribution of the substrate^28^,^33^. A mesh step of 7 nm is used throughout this study, yielding typically between 6000 and 9000 dipoles for a structure.

Figure 4c and g shows results from GDM simulations using the experimental parameters. For large antenna gaps, the arms are only weakly coupled and excitation along X and Y polarizations results in excitation of the longitudinal modes of the individual nanorods. The experimental and calculated *σ*_YY_ spectra both show optical resonances with wavelengths increasing from 1.0 *μ*m to 1.6 *μ*m as the vertical antenna length is increased from 180 nm to 330 nm. For completely uncoupled arms, excitation along A and B polarizations result in equal superpositions of the individual arm modes. With increasing coupling, the antenna arms are hybridized resulting in a difference between the *σ*_AA_ and *σ*_BB_ spectra. For both gaps under study, the A and B modes are different both in the experiment and the GDM model showing that coupling already exists for the largest gaps under study. However, for the smallest gaps the coupling is increased and the difference between *σ*_AA_ and *σ*_BB_ is pronounced. In this case, the long wavelength resonance is only present for illumination along the B-polarization.

Next to spectra taken at parallel polarizations, polarization conversion results in scattering cross sections in the perpendicular polarization direction. Figure 4d,e shows measured and simulated PC spectra for the cases of *σ*_YX_ and *σ*_BA_ with large gap. For weakly coupled arms, the Y → X PC results in overall low intensities for the different components. For the X → Y PC, the analytical model of Fig. 3 predicts zero intensity in absence of coupling. The remaining scattering intensity in the *σ*_YX_ PC is a result of the finite amount of coupling for these relatively large gaps. In comparison, B → A PC results in much higher intensities for the asymmetric antennas, consistent with the theoretical model calculations of Fig. 4e. Only for the symmetric case (red curve) *σ*_BA_ is very low, confirming the results of our analytical model. Results of the PC for more strongly coupled antenna arms are presented in Fig. 4h,i. The increased coupling strength results in a significant increase of the PC intensity in the Y → X direction for all antennas.

Qualitatively good agreement is obtained between the trends observed in the experimental and calculated spectra. Some differences can be attributed to the intrinsic variation of single-antenna experiments, while generally the calculated resonances are somewhat narrower and more defined than the experimental antenna resonances. We point out a systematic inaccuracy in the absolute cross sections of around a factor 2, which is similar to the level of agreement found in earlier works^6^ and which may reflect some inaccuracy in the assumptions underlying the calibration. However, relative magnitudes between antennas are in agreement and are not influenced by a systematic offset.

**Figure 5:**
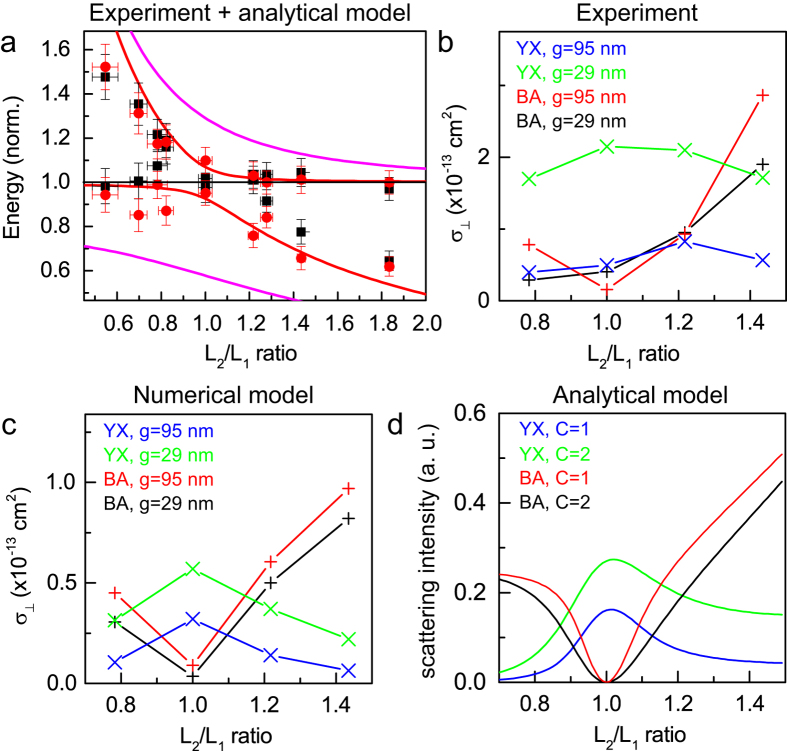
(**a**) Resonance frequency of the optical resonances measured on several individual antennas using spatial modulation spectroscopy normalized to the frequency of a single arm without coupling, *λ*_*sp*,1_. Black squares: gap *g* = 87 nm. Red circles: *g* = 29 nm. Solid lines are calculations using analytical model for coupling strength *C* = 1.5 (red lines) and *C* = 7 (magenta lines). (**b,c**) Maximum scattering cross sections for the perpendicular scattering polarization, *σ*_⊥_, for the cases of B → A and Y → X polarization conversion. Plotted are the cases of large gap (*g* = 95 nm) and small gap (*g* = 29 nm). Experimental values are plotted in (**b**), GDM calculations are shown in (**c**). Predictions of the analytical model are shown in (**d**) for coupling strengths *C* = 1 (blue, red) and *C* = 2 (green, black).

To further assess the agreement between experiment, numerical model and analytical theory, we compare in Fig. 5 experimental and calculated values for the mode dispersion and PC intensities. Figure 5a presents the resonance wavelengths measured on several individual antennas. In addition to the *L*_2_/*L*_1_ ratios of Fig. 4 we measured a number of other antenna combinations with asymmetry ratios in the range 0.5 - 1.8. The results are normalized to the resonance wavelength of a single arm of length *L*_1_ to allow direct comparison with the analytical model. The solid lines in Fig. 5a illustrate the resonance splittings obtained using the analytical model calculations for coupling coefficients *C* = 1.5 (red) and 7.0 (magenta). As expected for a coupled oscillator system, a stronger coupling, increases the spectral splitting between the antenna eigenmodes. The variation of *λ*/*λ*_*sp*,1_ with *λ*_*sp*,2_/*λ*_*sp*,1_ can be accurately reproduced by the analytical model for a coupling strength of *C* = 1.5.

Figure 5b–d compare the cross-sections for the maximum intensity scattered with polarization along the perpendicular direction, denoted as *σ*_⊥_, as a function of the asymmetry ratio *L*_2_/*L*_1_ for the experiment (b), the numerical model (c) and the analytical model (d). The data points in Fig. 5b,c correspond to values take from the spectra of Fig. 4. Figure 5d shows results from the analytical model of Section 1, which was refined by scaling the polarizability *α*_2_ proportional to the antenna length (i.e. the particle volume). This refinement was made to improve the agreement with the experimental geometry where the increased antenna length results in an increased resonance cross section. The analytical theory of Fig. 5d and the numerical model of Fig. 5c show good agreement in the observed trends. The experimental results show global trends that confirm the model, within the uncertainty given by the complexity of single-antenna experiments as seen in Fig. 4.

The difference between (B → A) and (Y → X) PC with *L*_2_/*L*_1_ ratio is clearly illustrated and confirms the validity of the PC model for antennas combining anisotropy and coupling. The numerical simulations clearly demonstrate that the maximum *σ*_YX_ is in all cases obtained for symmetric antennas. In comparison, PC along the B → A is zero for the symmetric case and increases strongly for asymmetric antennas. When coupling is introduced to the antennas, the B → A conversion becomes weaker while the Y → X conversion increases. This behaviour is in perfect agreement with the theoretical considerations in the context of our analytical model, reflecting the fact of antenna-mode driven polarization conversion for asymmetric antennas (B → A) and PC induced by mode hybridization due to coupling in the symmetric case (Y → X).

## Conclusion

In conclusion, we have investigated polarization conversion in plasmonic dimer nanoantennas. Using an analytical model for two coupled dipolar antennas based on field susceptibilities we showed, that polarization conversion efficiency can be tuned by carefully adjusting the asymmetry and coupling, both having an impact on the spectral splitting between the two arms, either by a simple change in the resonance frequency of the antenna arms or by hybridization of the modes due to coupling. In the case of coupled symmetric antennas, polarization conversion is most efficient along the *X* and *Y* directions and does not occur along the bonding and anti-bonding modes. Uncoupled asymmetric antennas, on the other hand, show efficient PC for incident polarizations along the diagonal directions corresponding to 45° and 135°. Finally we found, that by introducing coupling to asymmetric antennas, the angles of highest PC efficiency are tilted.

These results indicate that it is possible to control the scattering from L-shape antennas both via their structural morphology and interparticle interactions. The dependencies upon antenna morphology, excitation wavelength and polarization can be faithfully captured by a simple analytical model. Optical spectroscopy experiments performed on individual antennas are in good agreement with this model and with electrodynamical simulations based on the Green Dyadic Method. Our results show that optimal polarization conversion is obtained in the case of symmetrical antennas. They provide useful design rules for integration of these nanostructures in phase-discontinuity surfaces for applications like flat lenses or spiral waveplates.

## Methods

### Analytical two-oscillator model

The coupled set of equations is solved in the following way. Equations (5) and (6) can be rewritten in the following way:


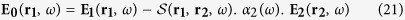


and


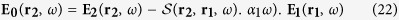


We define the supervectors 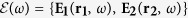 (resp. 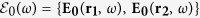) containing the total (resp. incident) electric field at the two particle locations. The complete linear system now takes the form:


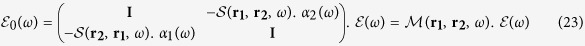


In the general case, the 6 × 6 matrix 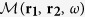 must be inverted in order to get the self-consistent electric field on both particles:


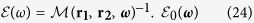


with


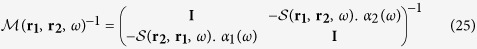



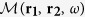
 has the following expression:


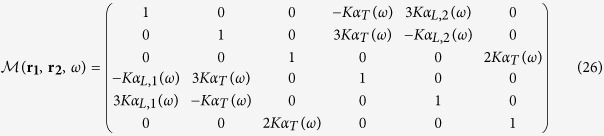


It can be noted from equation 26 that the magnitude of the electromagnetic coupling between the antenna arms depends upon the product of *K* by the polarizabilities. Therefore, in the following, we define the coupling constant *C* = *K*|*α*_*L*,1_(*ω*_*sp*,1_)|). *C* is dimensionless as the polarizabilities are homogeneous to a volume (CGS units). In the following, we focus on a spectral range far from the transverse resonance of the antenna arms and therefore assume that *α*_*T*_(*ω*) = 0. 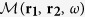 then becomes:


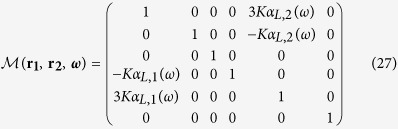


The (OX) and (OY)-components *E*_*x*_(***r***_**1**_, *ω*) and *E*_*y*_(***r***_**2**_, *ω*) of the total electric field can be computed after inversion of 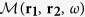. For an excitation polarized along (OX), we have:


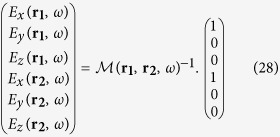


## Data Availability. 

All data supporting this study are openly available from the University of Southampton repository at http://dx.doi.org/10.5258/SOTON/404257.

## Additional Information

**How to cite this article:** Wiecha, P. R. *et al*. Polarization conversion in plasmonic nanoantennas for metasurfaces using structural asymmetry and mode hybridization. *Sci. Rep.* **7**, 40906; doi: 10.1038/srep40906 (2017).

**Publisher's note:** Springer Nature remains neutral with regard to jurisdictional claims in published maps and institutional affiliations.
